# “Clean” doping to advance 2D material phototransistors

**DOI:** 10.1038/s41377-022-00842-4

**Published:** 2022-06-06

**Authors:** Zhen Wang, Peng Wang, Weida Hu

**Affiliations:** State Key Laboratory of Infrared Physics, Shanghai Institute of Technical Physics, Chinese Academy of Sciences, 200083 Shanghai, China

**Keywords:** Photonic devices, Optical sensors

## Abstract

Doping is an essential element to develop next-generation electronic and optoelectronic devices and has to break the limit of specific steps during material synthesis and device fabrication. Here the authors reveal “clean” doping to enhance the electric and photoelectric performance of two-dimensional (2D) indium selenide (InSe) via a neutron-transmutation method for the first time, even after device fabrication.

Doping is the core issue of the semiconductor manufacturing process, which determines whether semiconductors can be widely applied and promote the leap-forward development of forefront high-density integrated devices or not. Early doping is called the effect of impurities in semiconductors and is known in crystal radio detectors and selenium rectifiers. Shelford Bidwell in 1885 and Bernhard Gudden in 1930 independently reported that the impurities in semiconductors would affect their properties. During World War II, John Robert Woodyard at Sperry Gyroscope Company formally developed a doping process in which tiny solids from the nitrogen column of the periodic table were added to germanium. Although the word “doping” is not used at that time, his US Patent described the “doping process” in detail.

With decades of development, controllable doping in silicon and germanium becomes more and more mature. Subsequently, thermal oxidation was used to achieve a high-quality Si–SiO_2_ system and the first metal-oxide-semiconductor field-effect transistor in 1960 so that the era of integrated circuits arrived. At present, the equipment with electronic and photonic devices have become an indispensable part of people’s daily life. However, as the device’s size scales down to the sub-10-nm technology, the technical challenge of device fabrication as well as the doping increase several-fold. Exploring alternative device geometries or new materials is ever more urgent.

Two-dimensional materials with novel properties, like atomically thin nature, strong light-mass interaction, tunable bandgaps, weak interlayer van der Waals force, and random stacking, not only provide a feasible way to solve the size limit of electronic devices based on traditional semiconductors, but also offer unprecedented freedom to design and fabricate new photonic devices. Doping engineering is a very significant approach to manipulating electronic and photonic characteristics of various 2D materials for advanced applications in neuromorphic hardware, logical circuits, and optoelectronic devices. To date, much effort has been made on achieving doping in 2D materials. Due to tunable electronic states in the two-dimensional plane, several research groups independently realized n-type and p-type doping in different 2D materials by electrostatic effect^1^, surface charge transfer^2^, and interlayer intercalation^3^. Jin et al. obtained a p-type 2D MoSe_2_ crystal in which Mo atoms are substituted by Nb atoms during material synthesis^4^. Furthermore, Wang et al. reported controllable thickness-modulated doping in a series of 2D materials, including wide and narrow bandgap materials^5^. Moreover, thickness-modulated heterojunction and avalanche photodetectors are successfully fabricated and exhibit high performance^6–8^.

Doping in 2D materials mentioned above is realized during crystal growth or post-growth process, which makes the doping occurs at specific steps. Moreover, some additional reagents are used in substitutional, surface charge transfer, and intercalation doping, which may bring in contaminants. To solve these problems, in the new issue of eLight, Guo et al. demonstrate a “clean” method to manipulate electric and photoelectric properties of 2D material via neutron-transmutation doping (NTD) for the first time, which is not limited by the semiconductor manufacturing process^9^. Since 2D layered InSe possesses a tunable bandgap and ultrahigh mobility (1000 cm^2^ V^−^^1^ s^−^^1^) at room temperature, the authors choose 2D InSe as the host material. NTD could be conducted on both material synthesis and device fabrication. After thermal neutrons irradiation on 2D InSe, the main nuclear reaction occurs in the indium atoms, as shown in Fig. 1a. The transmutation reaction is displayed by ${}_{49}^{115}{\rm{In}}(n,\gamma){}_{49}^{116}{\rm{In}} \to {}_{50}^{116}{\rm{Sn}}$ (*β*^-^ decay). The ^115^In isotope of InSe is transformed into the unstable ^116^In isotope. ^115^In isotope and ^116^In isotope are transmuted into ^116^Sn. Meanwhile, *γ* and *β*^-^ are emitted.

**Fig. 1:**
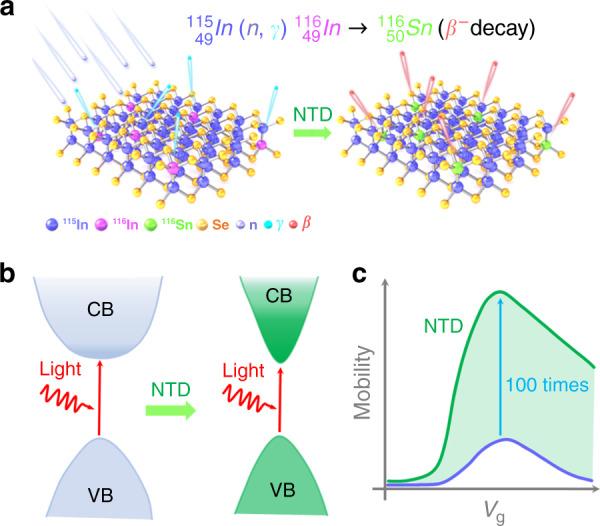
Electric and photoelectric properties of 2D layered InSe manipulated by a “clean” neutron transmutation doping. **a** Transmutation neutron doping (NTD) scheme for 2D layered InSe, including the capture of thermal neutrons and decay of *γ* and *β*^*-*^ particles. **b**, **c** Energy band structures and mobility in 2D layered InSe before and after transmutation neutron doping. CB and VB represent the conduction band and valence band, respectively

After the transmutation reaction, some In atoms in InSe are changed into Sn atoms. Sn atoms in InSe are in-situ substitutional atoms and lead to shallow donors. The charge density of the Sn atoms mainly contributes to the Sn–In bonds in Sn–InSe. The carrier concentration of Sn-doped InSe is approximately one order of magnitude higher than that of the nonirradiated samples. Moreover, the bandgap of InSe greatly decreases, which is very suitable for broadband photodetection, as shown in Fig. 1b. These obvious redshifts are also verified by the transient absorption spectra. In the meantime, the lifetime of carriers in Sn-doped InSe becomes longer. More importantly, the electron effective mass of Sn-doped InSe is smaller than that of the intrinsic InSe. This means that the electrons in Sn-doped InSe would possess a faster speed at the same bias voltage, compared with the pristine InSe. The carrier mobility in Sn-doped InSe field-effect transistors increases 100 times, shown in Fig. 1c. Excitingly, the response wavelength and responsivity of the Sn-doped InSe based phototransistor are broadened to 1064 nm and enhanced by about 50 times, respectively.

The proposed doping technique by Guo et al.^9^ can be applied in any step of the semiconductor manufacturing process and does not induce excessive reagents. NTD will accelerate the development of next-generation electronic and optoelectronic devices with high speed, low power, and small size. It may even restore some inoperative devices to increase life span, which will leave a lower cost of failure devices. In addition, NTD offers an intriguing tool to study and manipulate optical, electric, mechanical, and catalytic properties of materials.
